# Cellular and molecular alterations in neurons and glial cells in inherited retinal degeneration

**DOI:** 10.3389/fnana.2022.984052

**Published:** 2022-09-26

**Authors:** Natalia Martínez-Gil, Victoria Maneu, Oksana Kutsyr, Laura Fernández-Sánchez, Xavier Sánchez-Sáez, Carla Sánchez-Castillo, Laura Campello, Pedro Lax, Isabel Pinilla, Nicolás Cuenca

**Affiliations:** ^1^Department of Physiology, Genetics and Microbiology, University of Alicante, Alicante, Spain; ^2^Department of Optics, Pharmacology and Anatomy, University of Alicante, Alicante, Spain; ^3^Alicante Institute for Health and Biomedical Research (ISABIAL), Alicante, Spain; ^4^Aragón Institute for Health Research (IIS Aragón), Zaragoza, Spain; ^5^Department of Ophthalmology, Lozano Blesa University Hospital, Zaragoza, Spain; ^6^Department of Surgery, University of Zaragoza, Zaragoza, Spain; ^7^Institute Ramón Margalef, University of Alicante, Alicante, Spain

**Keywords:** retinal degeneration, cellular responses, oxidative stress, inflammation, reactive oxygen species

## Abstract

Multiple gene mutations have been associated with inherited retinal dystrophies (IRDs). Despite the spectrum of phenotypes caused by the distinct mutations, IRDs display common physiopathology features. Cell death is accompanied by inflammation and oxidative stress. The vertebrate retina has several attributes that make this tissue vulnerable to oxidative and nitrosative imbalance. The high energy demands and active metabolism in retinal cells, as well as their continuous exposure to high oxygen levels and light-induced stress, reveal the importance of tightly regulated homeostatic processes to maintain retinal function, which are compromised in pathological conditions. In addition, the subsequent microglial activation and gliosis, which triggers the secretion of pro-inflammatory cytokines, chemokines, trophic factors, and other molecules, further worsen the degenerative process. As the disease evolves, retinal cells change their morphology and function. In disease stages where photoreceptors are lost, the remaining neurons of the retina to preserve their function seek out for new synaptic partners, which leads to a cascade of morphological alterations in retinal cells that results in a complete remodeling of the tissue. In this review, we describe important molecular and morphological changes in retinal cells that occur in response to oxidative stress and the inflammatory processes underlying IRDs.

## Introduction

Inherited retinal dystrophies (IRDs) are a heterogeneous group of genetic disorders with different phenotypes and severity with a prevalence of 1 in 3,000 individuals (Sahel et al., 2015), which affect more than 2 million people worldwide (Berger et al., 2010). They include retinitis pigmentosa (RP, the most common form of rod dystrophy), Usher syndrome, Leber congenital amaurosis (LCA), or Stargardt disease (the most common form of cone dystrophy). Whichever their phenotype and pathogenesis are (rod- or cone-dominated, generalized degeneration or vitreoretinopathy), IRDs share common mechanisms at molecular level. Genetic variations affect the folding and/or function of different proteins, which will alter particular signaling pathways and trigger initially a specific cell-type death. In every case, all the retinal cells are progressively affected by a surrounding environment of increasing oxidative stress, inflammation, and cell death that will inevitably lead to cell death and to a gradual and irreversible vision loss.

The retina is exposed to high oxygen levels by the choroidal vascularization, which makes it more vulnerable to oxidative stress. The photoreceptors are one of the most metabolically active cells in the organism. High quantities of oxygen are needed for the phototransduction process, and the large cluster of mitochondria in the ellipsoid zone makes the photoreceptors great consumers of oxygen (Eshaq et al., 2014). These mitochondria generate great quantities of reactive oxygen species (ROS) (Kowaltowski et al., 2009). In a balanced situation, the ROS and reactive nitrogen species (RNS), generated as a result of cellular metabolism, can be neutralized or catalyzed, due to enzymatic antioxidants as copper–zinc and manganese superoxide dismutases, catalase, peroxiredoxin, glutathione peroxidase, and glutathione reductase and other non-enzymatic antioxidants as vitamins E, A, or C (Wang et al., 2021). Low levels of reactive oxiygen and nitrogen species (RONS) are needed to the control of cell signaling processes of key redox-sensitive residues in regulatory proteins as MAPK or PI3K/Akt and also the regulation of gene expression, transcription factors, and epigenetic pathways (Moldogazieva et al., 2018). But when an imbalance between RONS formation and removal occurs, the oxidative stress drives to the oxidation of proteins, lipids, and DNA and the activation of inflammation and cell death pathways. Photoreceptors are extremely sensitive to high RONS levels and lipid peroxidation, due to the large surface area of membranous disks enriched with polyunsaturated fats (Winkler et al., 1999; Valko et al., 2007; Panfoli et al., 2012; Wang et al., 2021) and will eventually die.

Oxidized molecules can trigger microglial activation, changing from a protective function in which they remove cellular debris and secrete protective trophic factors and cytokines, to an activated state in which they secret pro-inflammatory cytokines, chemokines, trophic factors, and other mediators, initially with a repairing goal but that, in a context of retinal degeneration, when the harmful stimulus persists and the microglial activation remains, contribute to chronic inflammation and cell death (Yoshida et al., 2013a,b). Also, the gliosis of astrocytes and Müller cells, which is activated in the early stages of the degenerative process, has initially a neuroprotective effect on the retina, as they increase the expression of cytoprotective factors, and restores neurotransmitter balance and ion water concentration, but a chronic gliosis exacerbates the neurodegeneration [reviewed in (Cuenca et al., 2014)].

After retinal neuron cells start to dye, pre- and/or post-synaptic inputs are lost, and the still surviving cells try to maintain functionality by stablishing new synaptic contacts. These changes induce morphological changes and a remodeling process that affects all the tissue structure, which is disease-independent (Cuenca et al., 2014). In this work, we review the retinal changes induced by oxidative stress and inflammation in the degenerating process of IRD, detailing the cellular and molecular responses.

## Cellular responses in the inherited retinal dystrophies in retinal pigment epithelium, photoreceptors, and glia

**FIGURE 1:**
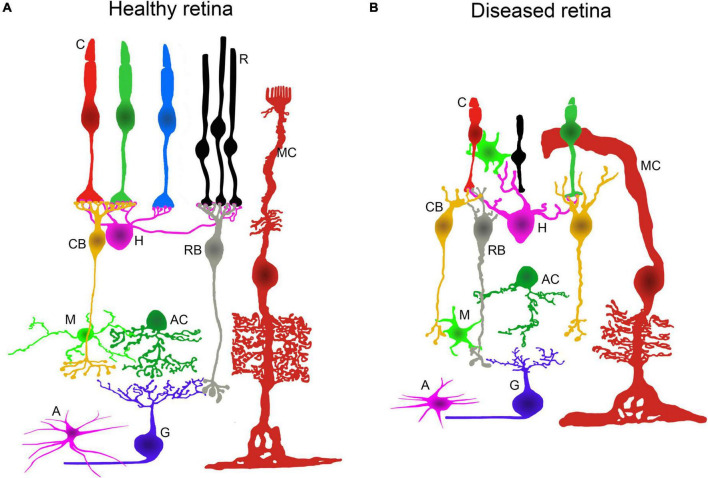
Retinal alterations associated to IRDs. Schematic representation of the retinal neurons and glial cells in healthy and diseased retinas. **(A)** Normal morphology and layered architecture of the mammalian retina in physiological conditions. **(B)** During retinal neurodegenerative processes, changes in morphology and connectivity take place. There is a loss of rods and major morphological alterations in cones. The dendrites from horizontal and bipolar cells degenerate. There is a generalized retraction and loss of axons, dendrites, and synaptic connections in the inner retinal neurons. Müller cells, microglia, and astrocytes display an activated state and undergo morphological changes. C: cone photoreceptor; R: rod photoreceptor; MC: Müller cell; CB: cone bipolar cell; H: horizontal cell; RB: rod bipolar cell; M: microglial cell; AC: amacrine cell; A: astrocyte; G: ganglion cell.

Molecular mechanisms underlying hereditary neurodegenerative diseases of the retina produce aberrant changes in the function and morphology of retinal cells (Marc and Jones, 2003; Jones et al., 2012, 2016; Cuenca et al., 2014; Strettoi, 2015; Pinilla et al., 2016; Pfeiffer et al., 2020). Retinal cell dysfunction is accompanied, in most cases, by photoreceptor cell death, neurodegeneration of the inner retina, and tissue remodeling promoting blindness. These responses, which occur during the retinal degenerative process, have common features in neurons and glial cells. The main alterations in cell morphology, metabolism, and protein expression (Cuenca et al., 2020; Pfeiffer et al., 2020), together with the retinal vasculature atrophy that occurs in IRDs, are illustrated in Figure 1.

### Retinal pigment epithelium responses

The retinal pigment epithelium (RPE) forms the outer blood-retinal barrier and is directly implicated in retinal homeostasis, including transport of nutrients and waste products, photoreceptor outer segment shedding, secretion of essential proteins and growth factors, light absorption, photooxidation protection, and the recycling of the chromophore 11-cis-retinal to conform the visual pigment for the visual cycle (Strauss, 2005). As mentioned earlier, several mutations that cause retinal dystrophies are found in RPE genes causing primary RPE dystrophies (Daiger and The University of Texas Health Science Center, Houston). Some of these mutations are localized in genes that encode the necessary proteins for the visual chromophore recycling and its incorporation into photoreceptors. The first step of the visual cycle occurs in RPE and involves the conversion of the all-trans-retinol (vitamin A) to all-trans-retinyl ester by lecithin retinol acyl transferase (LRAT). The all-trans-retinyl ester is converted to 11-cis-retinol by the retinal pigment epithelium-specific protein 65-kDa (RPE65) isomerase to be later oxidized to 11-cis-retinal by a dehydrogenase enzyme. Finally, the visual chromophore is transferred to rod and cone outer segments through the activity of the cellular retinaldehyde-binding protein (CRALBP). In photoreceptors, 11-cis-retinal is combined with opsins forming the visual pigment (e.g., rhodopsin). Mutations in the genes that encode CRALBP are responsible of retinitis punctata albescens, and *LRAT* and *RPE65* mutations have been described in autosomal recessive RP forms and LCA, among others (Daiger and The University of Texas Health Science Center, Houston). Also, RP has been directly related to mutations that compromise other RPE functions, and it is one of the five clinical forms of “bestrophinopathies,” caused by over 200 mutations in *BEST1* (Johnson et al., 2017). Mutations in *BEST1*, which encodes the ion channel bestrophin 1, or mutations in *MERTK*, which encodes the MER tyrosine kinase membrane receptor, alter the shedding of photoreceptor outer segments and intracellular calcium signaling (Johnson et al., 2017; Audo et al., 2018). Studies in Royal College of Surgeons’ (RCS) rats with *Mertk* gene mutations revealed that Mertk is also required for the apical expression of glucose receptors by RPE. Likewise, a decrease in glucose metabolism has also been described in other models of RP with *RHO* mutations, such as P23H pigs and mice (Wang et al., 2019).

**FIGURE 2:**
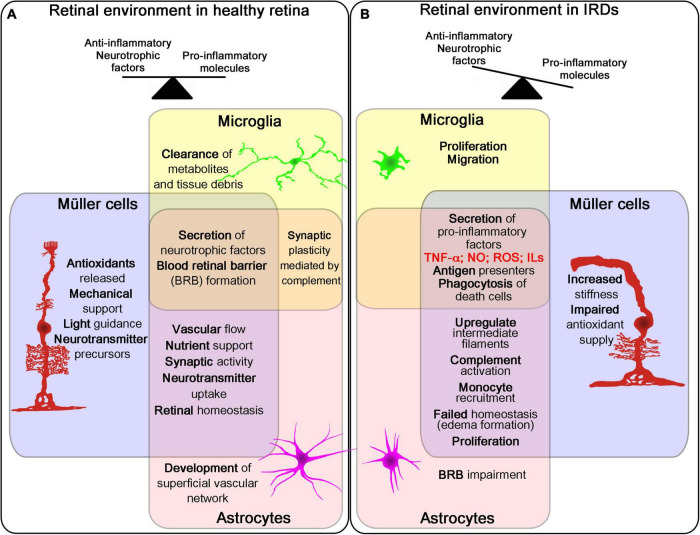
Role of retina glial cells in IRDs. Schematic representation of the three glial cell types in the retina (Müller cells, microglia and astrocytes) and their main functions and changes in healthy and disease retina. The three cells work together in different processes affecting retinal homeostasis in healthy retina **(A)**. During the course of retinal degeneration, these cells secrete pro-inflammatory factors and promote proliferation, migration and phagocytosis, increasing inflammatory signals in the tissue **(B)**.

Although these mutations compromise different functions of RPE, the cellular response shows a common pattern characterized by the loss of pigmentation, cell enlargement, vacuolization, loss of cell-to-cell contact, loss of apical microvilli and basal infoldings, multinucleation and migration to subretinal space, which ultimately leads to the disruption of the layer, photoreceptor disorganization, and retinal degeneration (George et al., 2021). Similar responses have been described in primary photoreceptor dystrophies where RPE dysfunction appears as a secondary sign of the disease (da Cruz et al., 2007). The inflammatory environment in the outer retina in IRDs promotes the blood-retinal barrier breakdown through the impairment of the RPE tight junctions (Napoli et al., 2021). The decrease of essential proteins such as zonula occludens-1 (ZO-1) is strongly related to the inflammatory and oxidative events triggered in these diseases (Napoli et al., 2021). Even more, this outer blood-retinal barrier breakdown has been described as a cause of visual acuity loss, together with the photoreceptor death in RP (Vinores et al., 1995). As these biological responses can be promoted by mutations in RPE-related genes or by changes in the extracellular and intracellular environment (due to the inflammatory and oxidative stress process activated by the disease), the balance between the pro-survival and cell death activated pathways is essential to maintain retinal health (Figure 2).

**FIGURE 3:**
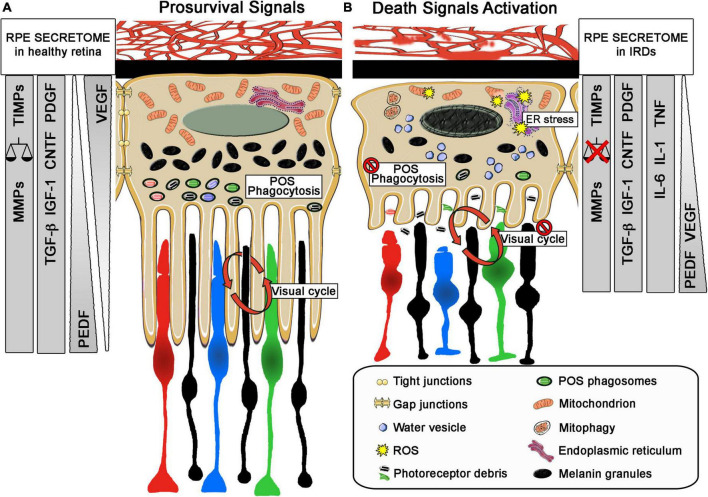
Role of retinal pigment epithelium (RPE) in IRDs. Schematic representation of main morphology and secretome profile changes of the relationship between RPE and photoreceptors in healthy retina and during retinal degeneration. **(A)** In healthy retina, RPE exert critical roles related to photoreceptor survive, as phagocytosis of photoreceptor outer segments (POS), visual cycle and forming the outer blood-retinal barrier and secreting neurotrophic factor such as VEGF from the basolateral to apical part of the RPE cell. In addition, the selective secretion of other neurotrophic factors and molecules support these roles and maintain the retinal homeostasis. **(B)** Under pathological conditions RPE cells change their morphology with shorter microvillis, less POS phagosomes and melanin granules and a diminution of the tight junction between them. They also present a disruption of their normal functions, failing in their POS phagocytosis and in the visual cycle role. They exert a change in their apical and basal secretome profile, increasing pro-inflammatory molecules or even changing the polarized secretion of VEGF. The injury stimuli on these cells can trigger cell stress increasing mitophagy, ER stress that can even disrupt the basic functions needed for the survival of photoreceptors. In both sizes, changes in their secretome profile are explained, including changes on the secretion size; VEGF in healthy retina is mainly secreted in the basal size of the RPE cell and will change to the apical size with PEDF. Other neurotrophic factors will also change. MMPs, matrix metalloproteinases; TIMPs, matrix metalloproteinases inhibitors; TGF β, transforming growth factor-β; IGF1, insulin-like growth factor-1; CNTF, ciliary neurotrophic factor; PDGF, platelet-derived growth factor; VEGF, vascular endothelial growth factor; CNTF, ciliary neurotrophic factor; PEDF, pigment epithelium-derived factor; IL1, interleukin 1; IL6, interleukin 6; TNF, tumor necrosis factor; ER, endoplasmic reticulum; POS, photoreceptor outer segments; ROS: reactive oxygen species.

Given its functions and anatomical location, RPE is subjected to biomolecule photooxidation, high accumulation of phagosomes, lysosomes, lipid-protein aggregates (such as lipofuscin), and high oxygen tension, which is intensified in retinal dystrophies with loss of photoreceptors (Sparrrow et al., 2010). Accordingly, the mechanisms promoted by RPE cells to counteract the oxidative stress and inflammation involve not only enzymatic defense (Wunderlich et al., 2010), but also the synthesis and release of antioxidant and anti-inflammatory molecules and pro-survival growth factors (Meyer et al., 2019; Martinez-Gil et al., 2020). The enzymatic activity of superoxide dismutase (SOD), glutathione peroxidase (GPX), catalase (CAT), peroxiredoxins (PRDXs), and other antioxidant enzymes is essential in RPE to buffer ROS (Pintea et al., 2011; Fang et al., 2016). These enzymes, together with crystallins, which are more abundant in RPE than in neural retina, contribute to the cellular reducing power (Kannan et al., 2016; Álvarez-Barrios et al., 2021). Additionally, impairment of the transport of metallothioneins (free radical scavengers) has also been related to photoreceptor degeneration in RCS rats (Wunderlich et al., 2010). This endogenous enzymatic defense has been analyzed in aqueous humor and serum samples of patients with RP (Martínez-Fernández de la Cámara et al., 2013; Ishizu et al., 2019; Ertan et al., 2021). Martínez-Fernández de la Cámara et al. reported a reduction in antioxidant capacity and, particularly, in the activity of SOD3 in both ocular tissue and peripheral blood, concomitant with an increase in nitroxidative stress (Martínez-Fernández de la Cámara et al., 2013). These results are in accordance with those of Ertan et al. in serum samples of patients with RP, as they also described an imbalance between oxidant status and antioxidant defense (Ertan et al., 2021). More recently, this decrease of SOD3 activity in the serum of patients with RP has been correlated with vision loss (Ishizu et al., 2019). It is well known that RPE secretes an array of proteins and growth factors such as FGFs, TGF-β, insulin-like growth factor-I (IGF-I), CNTF, and platelet-derived growth factor (PDGF), which are essential for the development and maintenance of retinal health (Strauss, 2005). The forced upregulation or exogenous administration of some of these factors has been tested as possible neuroprotective therapies for retinal dystrophies (Dias et al., 2018; Martinez Velazquez and Ballios, 2021). In some cases, their release is dependent on the polarization state of the cells. For instance, vascular endothelial growth factor (VEGF) is secreted on the basolateral side, whereas PEDF release occurs on the apical side. Under oxidative stress, the release of VEGF increases on the apical side to exert, together with PEDF, its neuroprotective action on the neural retina (Bouck, 2002). Beyond growth factors, the RPE secretome also includes inflammation-related molecules such as members of the interleukin family (IL-1 and IL-6) and TNF-α. Matrix metalloproteinases (MMPs) together with their inhibitors (TIMPs), which are crucial for retinal matrix stabilization and for the shedding of photoreceptor outer segments (Kay et al., 2013), can be modified in an RPE inflammatory and oxidative environment (Lu et al., 2020) (Figure 3). Recently, Bing et al. described an increase of IL-6, PDGF, and some MMPs in the aqueous humor of patients with RP and established a correlation between this increase and the appearance of cataracts (Lu et al., 2020).

Nuclear factor erythroid-2-related factor 2 (Nrf2), neuroprotectin D1 (NPD1), and glutathione (GSH) are considered as the major antioxidant response regulators in RPE. Under oxidative stress stimuli, Nrf2 translocates to the nucleus and binds to antioxidant response elements (AREs) of target genes to induce their expression; these include SOD and GSH-PX enzymes (Zhang et al., 2013). The lipid mediator NPD1, which is endogenously synthesized by RPE from DHA, potently inhibits apoptosis by attenuating the expression of pro-apoptotic proteins such as Bax and Bad and also reduces the levels of pro-inflammatory mediators such as NF-κB and cyclo-oxygenase-2 (COX-2) (Bazan, 2006). The phosphoinositide 3-kinase pathway (PI3K/Akt) and the MAPK family are important mediators of the protective signaling against oxidative stress (Resta et al., 2007).

A combination of *in vivo* and *in vitro* studies in different experimental models of retinal degeneration revealed that RPE dysfunction could be countered by upregulating Nrf2 (Han et al., 2020), even in RP (Wu et al., 2021). The activity of PI3K/Akt, ERK1/2, p38-MAPK, and GSK3β is required for Nrf2 activation and to, ultimately, inhibit photoreceptor cell death [124,125]. In a similar manner, the activity of PI3K/Akt and MAPKs is also needed for the increase in expression of the neurotrophic factors PEDF and VEGF (Resta et al., 2007). The antioxidant (reducing) power of GSH is due to its free-radical scavenging capacity to neutralize lipid peroxides, H_2_O_2_, and by its conjugation to small molecules, proteins, or lipids (Plafker et al., 2012). In the retina, the efficiency of the GSH redox system diminishes as the neurodegeneration progresses and is also age-dependent (Sreekumar et al., 2021). This can be explained by the finding that Nrf2, which also diminishes under the same conditions, is necessary for the GSH synthesis (Plafker et al., 2012). Moreover, *in vitro* studies suggest that GSH reduction promotes the senescence and death of RPE cells through ferroptosis and autophagy (Sun et al., 2018). The activation of these processes is directly related to lipid peroxidation and 4-hydroxynonenal (4-HNE) accumulation under oxidative stress conditions. Finally, the implication of autophagy in the RPE goes beyond photoreceptor outer segment recycling, as it has been described as both a survival and a cell death mechanism. More studies are needed to clarify its role on retinal dystrophies (Intartaglia et al., 2021).

### Photoreceptor responses

The death of photoreceptors is the main cause of blindness in IRDs, with oxidative stress and inflammation recognized as the major contributors. In total, two main types of mutations causing retinal dystrophies can be distinguished: those that first cause rod degeneration and then cone damage, as found in RP, LCA, or syndromic RP such as Usher syndrome; and those that affect only cones, such as Stargardt disease (Sancho-Pelluz et al., 2008; Arango-Gonzalez et al., 2014). In pathologies such as RP, in which the progressive loss of rods is followed by the gradual death of cones, the precise mechanisms driving the secondary death of cone photoreceptors remain poorly understood. Many hypotheses have been formed to explain this. One of the most accepted states that, after the death of rods and the loss of its high oxygen-demand, there is an increase in oxygen levels in the retina, which induces cone degeneration by oxidative damage (Komeima et al., 2006).

**FIGURE 4:**
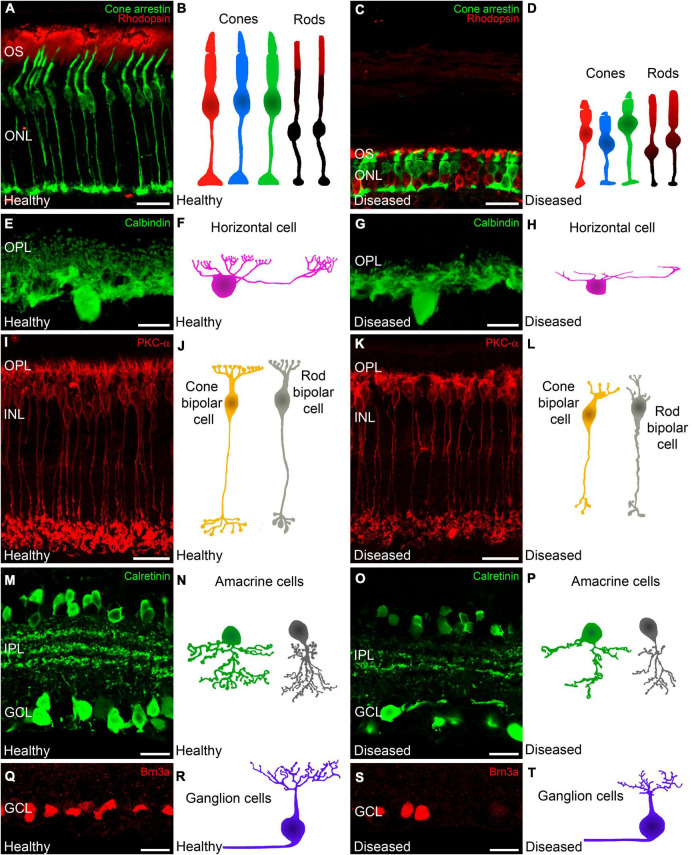
Changes in photoreceptors and inner retinal neurons during retinal degeneration. Retinal cross-sections immunostained with different retinal cell markers followed by a schematic drawing of each cell in a healthy mouse retina and in a retina from an IRD model. Photoreceptors **(A,C)** immunolabeled against cone arrestin (cone cells in green) and rhodopsin (outer segments of rods, red). **(A,B)** Normal morphology of cones and outer segments of rod photoreceptors. **(C,D)** In IRD affected retinas, cones show shortened and swollen outer and inner segments as well and a reduced axon length. Moreover, rods display an abnormal distribution of rhodopsin. **(E,G)** Horizontal cells immunostained against calbindin. **(E,F)** Normal morphology of horizontal cells showing the tips of the terminal branches from their axons and dendrites. **(G,H)** In IRD affected retinas, horizontal cells show shortened dendrites and axons accompanied with loss of synaptic terminals. **(I,K)** ON rod bipolar cells immunostained against PKCα. **(I)** Shows the normal morphology of ON rod bipolar cells whereas **(K)** shows retraction of bipolar cell dendrites and axons in IRD conditions. **(J,L)** Schematic representations of cone and rod bipolar cells in healthy **(J)** and diseased **(L)** conditions. **(M,O)** Calretinin immunostaining showing subpopulations of amacrine and ganglion cells as well as 3 immunoreactive plexuses in the IPL. **(M)** Shows the normal cell density and plexus structure and **(O)** Shows the loss of amacrine and ganglion cells with degeneration together with the loss of dendrites and synaptic contacts in the IPL. **(N,P)** Schematic depictions of representative amacrine cells in healthy conditions **(N)** and affected by retinal degeneration **(P)**. **(Q,S)** Brn3a immunostaining to allow the identification of retinal ganglion cell somata. Normal ganglion cell density in healthy retinas **(Q)** and ganglion cell loss in IRD affected retinas **(S)**. **(R,T)** Schematic depiction of normal ganglion cell morphology in healthy retinas **(R)** and dendritic alterations in diseased retinas **(T)**. OS: outer segment; ONL: outer nuclear layer; OPL: outer plexiform layer; INL: inner nuclear layer; GCL: ganglion cell layer. Scale bars: 20 μm **(A,C,I,K,M,O,Q,S)**; 10 μm **(E,G)**.

**FIGURE 5:**
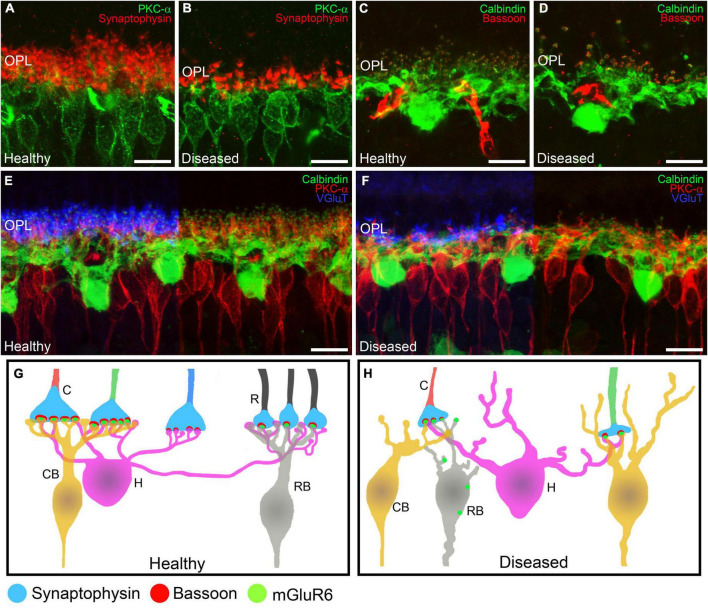
Alterations in retinal cell connectivity in IRDs. **(A–F)** Retinal cross-sections immunostained with different retinal cell markers in a healthy mouse retina and in a retina from an IRD model. **(A,B)** Shows the dendrites of ON rod bipolar cells (PKCα, green) making contacts with photoreceptor axon terminals (synaptophysin, red). **(A)** Average synaptic contact density and morphology of rod bipolar cell dendrites in the OPL in healthy retinas. **(B)** Degeneration of ON rod bipolar cell dendrites and loss of photoreceptor synaptic terminals in IRDs retinas. **(C,D)** Horizontal cells (calbindin, green) being part of the ribbon synapse (Bassoon, red) along with photoreceptors. **(C)** Average synaptic contact density and morphology of horizontal cell dendrites in healthy retinas. **(D)** Loss of horizontal cell dendrites and synaptic ribbons from photoreceptors in IRDs. **(E,F)** Horizontal cells (calbindin, green) and ON rod bipolar cells (PKCα, red) making synaptic contacts with axonic terminals from photoreceptors (VGluT, blue). **(E)** Average synaptic contact density and morphology of horizontal and ON rod bipolar cell dendrites in healthy retinas. **(F)** Dendritic loss of horizontal and ON rod bipolar cells and degeneration of synaptic connections with photoreceptor axonic terminals. **(G,H)** Schematic depiction of the normal synaptic connections from rods and cones to bipolar cells in the OPL. Synaptophysin is located in the presynaptic terminals from rods and cones. Bassoon is part of the synaptic ribbon and mGluR6 is localized in the synaptic tips of the bipolar cell dendrites. During synaptic remodeling induced by IRDs **(B)** there are alterations in the synaptic contacts from the OPL. Rod bipolar cells make contacts with cone terminals and mGluR6 is shown delocalized around the somas of bipolar cells. OPL: outer plexiform layer; C: cone; R: rod; CB: cone bipolar cell; H: horizontal cell; RB: rod bipolar cell. Scale bars: 10 μm.

In the early stages of IRDs, photoreceptor stress triggers a series of signaling cascades that activate cell death pathways, causing progressive photoreceptor loss. Initially, however, photoreceptor cell morphology and function are preserved, and opsin delocalization is one of the earliest histological indicators of pathology (Roof et al., 1994; Martinez-Navarrete et al., 2011; Kutsyr et al., 2020). When cone photoreceptor cells begin to deteriorate, they present shortened and swollen inner segments in addition to shortened outer segment length (Figures 1, 4A–D) (Martinez-Navarrete et al., 2011; Kutsyr et al., 2020). Also, axon length is progressively reduced until pedicles emerge directly from the soma (Campello et al., 2020; Kutsyr et al., 2020). Synaptic terminals from the remaining photoreceptors then start to degenerate with a delocalization of markers of pre- and post-synaptic profiles (Bassoon, synaptophysin, or mGluR6) (Figures 5A–H) (Cuenca et al., 2005).

Neuronal death in degenerative diseases is traditionally related to apoptosis; however, there is strong evidence that photoreceptor death is not exclusively directed by apoptosis in retinal dystrophies, and alternative non-apoptotic cell death pathways prevail (Sancho-Pelluz et al., 2008; Arango-Gonzalez et al., 2014; Pinilla et al., 2022). Hence, a wide range of apoptotic and non-apoptotic death mechanisms are activated in almost all IRDs that affect photoreceptor cells, including parthanatos, calpain-dependent cell death, or ferroptosis, among others. Although these main death pathways can be common in all diseases, the first death signal in photoreceptor cells is closely linked to the specific gene mutation causing each disease. Along this line, Viringipurampeer et al. (2016) demonstrated that inflammasome activation and pyroptosis have a major role in the secondary death of cones in the P23H (*rhodopsin* mutation) rat model of RP. This contrasts to the findings of Murakami et al. in rd10 (*phosphodiesterase 6b* mutation) mice, who showed that necroptosis is the major mechanism of cone but not rod photoreceptor cell death (Murakami et al., 2012). Thus, various cell-death pathways are triggered in different RP genotypes, and different mutations in the same gene can drive photoreceptor cell death with different phenotypes by disturbing different biological processes. Indeed, different mutations in *rhodopsin* can generate diverse cellular disturbances related to protein misfolding and endoplasmic reticulum (ER) stress, instability of photoreceptor outer segments, disrupted vesicular traffic, or altered outer segment formation (Athanasiou et al., 2018).

Among the essential functions that can be disturbed in photoreceptor cells during the course of degeneration include the phototransduction cascade, metabolic supplies, Ca^2+^ and cGMP homeostasis, and mitochondrial or ER function. Thus, mitochondria and the ER are the main points of integration of death signals (Kutluer et al., 2020). The ER is crucial for protein biosynthesis and folding, lipid biosynthesis, and Ca^2+^ homeostasis. ER stress activates pathological signaling pathways of oxidative stress, inflammation, and immune responses and plays a crucial role in retinal dystrophies of different etiologies (Sizova et al., 2014; Zhang et al., 2014; Fernández-Sánchez et al., 2015; Wei et al., 2021). Using the mouse rd1 (*phosphodiesterase 6b*) model, Jiang et al. recently showed that mitochondrial dysfunction is the earliest event in rod cell death in RP and suggested that mitochondria are the integrative node in the cellular responses to the genetic mutations that cause retinal dystrophy (Jiang et al., 2022). Mitochondrial dysfunction was triggered by a dysregulation in calcium signaling (Ke et al., 2021).

Calcium homeostasis is essential for photoreceptor function and survival (Krizaj and Copenhagen, 2002; Vinberg et al., 2018). Ca^2+^ modulates phototransduction in rods and cones through the Ca^2+^-binding proteins guanylyl cyclase-activating proteins 1 and 2 (GCAP1/2), which regulate the synthesis of cGMP by retinal membrane guanylyl cyclases (RetGC1 and RetGC2) (Vinberg et al., 2018). In photoreceptor cells, Ca^2+^ enters through cGMP-gated channels (CNGs) in the outer segments, and through L-type calcium channels in the cell body and the synaptic terminal, and exits through rod- and cone-specific Na^+^/Ca^2+^, K^+^ exchangers (Schnetkamp et al., 2014; Vinberg et al., 2018; Das et al., 2021).

The intracellular Ca^2+^ level is dependent on its entry and exit from/to the extracellular space, and on the buffering exerted by the ER and mitochondria. In this sense, mitochondria located in the inner segments of photoreceptors may also affect the Ca^2+^ level in the outer segments (Giarmarco et al., 2017). Mutations driving the excessive entry of Ca^2+^ or its reduced extrusion in photoreceptors lead to retinal degeneration. Thus, mutations in *PDE6* disturbing the hydrolytic activity of the protein markedly affect the intracellular levels of cGMP and hence lead to the opening of CNG channels and increased Ca^2+^ entry. These mutations are related to RP in humans, and the severity of retinal degeneration correlates with the degree of impairment of PDE6 activity (McLaughlin et al., 1993; Vinberg et al., 2018). Also, mutations in GCAP proteins are associated with LCA, macular dystrophies, and other cone-rod dystrophies (Downes et al., 2001; Wilkie et al., 2001; Nishiguchi et al., 2004; Jiang et al., 2005; Sokal et al., 2005; Vinberg et al., 2018), and mutations that cause misfolding of rhodopsin are associated with increased Ca^2+^ levels (Shinde et al., 2016). Photoreceptor dysfunction is also associated with the loss of Ca^2+^ homeostasis in patients with Duchenne muscular dystrophy (Krizaj and Copenhagen, 2002) a genetic disorder caused by a defective expression of the cytoskeletal protein dystrophin (Dp427) which is characterized by progressive muscle degeneration, and different forms of cognitive impairment, neurological and autonomic disorders, and specific visual defects. Increasing evidence shows that high cGMP levels are directly related to photoreceptor cell death in retinal dystrophies (Power et al., 2020). In addition to *PDE6* mutations, elevated cGMP levels result from cyclic nucleotide-gated channel mutations, in which Ca^2+^ levels are low. Hence, dysregulation of cGMP is likely to have a much more relevant role in photoreceptor death than previously thought, and it has been suggested that therapeutic strategies should focus also on cGMP signaling pathways (Paquet-Durand et al., 2019).

New relevant players in the photoreceptor degenerative process of retinal dystrophies have recently emerged, including sterile alpha and toll/interleukin-1 receptor motif-containing 1 (SARM1). SARM1 is a multidomain NAD + nucleosidase belonging to the toll/IL-1 receptor (TIR) domain-containing superfamily with a recognized relationship with axon degeneration and cell death (Osterloh et al., 2012). Recently, Ozaki et al. showed that Sarm1 has a pro-degenerative role in a rhodopsin-knockout mouse model of RP, as deficiency for *Sarm1* promotes photoreceptor cell survival (Ozaki et al., 2020). SARM1 is activated by oxidative stress (Ozaki et al., 2020), which triggers the loss of cellular ATP, mitochondrial depolarization, calcium influx, loss of membrane permeability, and cell death (Ko et al., 2021). The finding that SARM1 is expressed in photoreceptors and that it is related to rod and cone degeneration in IRDs makes it an interesting candidate for therapy (Ozaki et al., 2020). Recent gene therapy-based experiments using a mouse model of axon degeneration have shown that inhibiting Sarm1 blocks the degeneration, supporting this protein a promising target for future pharmacological and gene-based therapeutic strategies (Geisler et al., 2019). Histone deacetylates have been also suggested as potential therapeutic targets. All retinal degenerative diseases present with photoreceptor cell death accompanied by the increased activity of several enzymes. Among them, histone deacetylates appear to have a major role, as their inhibition increases cone survival, independently of which photoreceptor type is first affected (Sancho-Pelluz et al., 2010; Trifunović et al., 2016, 2018).

Recent work from our laboratory highlighted the relevant changes in fatty acids, especially docosahexaenoic acid (DHA), in rd10 mice during the degenerative process (Ruiz-Pastor et al., 2021). Fatty acids are an essential part of photoreceptor membranes, and of their cell integrity and are involved in the phototransduction cascade and change to their levels during retinal degeneration. DHA has been used in clinical trials to treat both inherited and neurodegenerative retinal diseases.

Photoreceptor degeneration, with the subsequent loss of synaptic input, triggers a series of rewiring events. In some models of IRDs, rod bipolar cells exhibit extensive dendritic sprouting and form ectopic synapses with cone terminals (Figures 1, 4I–L) (Peng et al., 2000, 2003; Marc et al., 2003; Pfeiffer et al., 2020). Importantly, in models of RP, this process starts before the complete loss of rod photoreceptors and, therefore, before cones begin to degenerate (Pfeiffer et al., 2020). Later in the degenerative process, bipolar cells experience complete deafferentation from photoreceptor loss. Bipolar cell remodeling comprises disorganization and retraction of bipolar cell dendrites and synaptic delocalization of iGluRs and mGluR6 receptors into bipolar cell bodies and axons (Figures 5G,H) (Strettoi and Pignatelli, 2000; Jones et al., 2011; Gayet-Primo and Puthussery, 2015). At this stage, however, iGluRs are still functional in amacrine and ganglion cells (Marc et al., 2007). Apical neurites from amacrine cells also make aberrant synapses with horizontal cells, likely altering lateral inhibition networks (Pfeiffer et al., 2020). Ultimately, when there is a complete loss of afferent input, horizontal, amacrine, and ganglion cell populations degenerate (Figures 1, 4E–H, M–T) and extend anomalous neurites forming large tangles of GABAergic, glycinergic, and glutamatergic processes termed microneuromas (Jones et al., 2003; Jones and Marc, 2005).

### Glial responses

The correct function of the retina requires an exquisitely regulated cellular network. The main cells responsible for maintaining retinal homeostasis are the microglia and the two major types of retinal macroglia: astrocytes and Müller cells.

#### Microglia

The microglia population in the retina has an important role in IRDs. Beyond its function of immune surveillance and defense, as the resident phagocytes in the retina, it is widely accepted that the microglial population is a key player in the fate of photoreceptors in retinal dystrophies (Akhtar-Schäfer et al., 2018; Wooff et al., 2019; Karlen et al., 2020). In the healthy retina, microglia are protective. With a ramified morphology, they continuously scan the surrounding area, clearing metabolic products and tissue debris. They also secrete anti-inflammatory cytokines, antioxidants, and growth factors, which contribute to the protection of photoreceptors. Chief among them are brain-derived neurotrophic factor (BDNF), ciliary neurotrophic factor (CNTF), glial cell line-derived neurotrophic factor, nerve growth factor (NGF), neurotrophin-3, and basic fibroblast growth factor (bFGF) (Langmann, 2007). When activated, microglia change their morphology to an amoeboid form, allowing them to move toward the damaged zone, proliferate, and exert enhanced phagocytic activity. In this activated state, microglia secrete a plethora of pro-inflammatory cytokines including TNF-α, IL-1β, and IL-6, in addition to ROS and nitrogen reactive species (RNS), which can induce photoreceptor death and aggravate the degenerative process (Roque et al., 1999; Langmann, 2007; Li et al., 2021) (Figure 2). Microglia can adopt a multitude of phenotypes depending on the environmental signals, with different expression patterns, and exert neuroprotective or neurotoxic effects depending on the pathological context (Schwartz et al., 2006; Hanisch and Kettenmann, 2007; Michelucci et al., 2009; Li et al., 2021; Lier et al., 2021).

To date, the dual role of microglia in retinal neurodegenerative diseases as both neuroprotective and pro-inflammatory is not fully understood, but it is accepted that they have a pivotal role in both the initiation and propagation of neurodegenerative processes (Madeira et al., 2015). Activation of microglia has been demonstrated in neurodegenerative CNS disorders such as Alzheimer’s or Parkinson’s disease, amyotrophic lateral sclerosis, and multiple sclerosis (Polazzi and Monti, 2010), and in most, if not all, retinal diseases, including traumatic damage (Harada et al., 2002; Zhang et al., 2005) and degenerative processes as glaucoma (Johnson and Morrison, 2009; Bosco et al., 2011), age-related macular degeneration (Ardeljan and Chan, 2013), DR (Kinuthia et al., 2020), and IRDs (Karlstetter et al., 2010; Massengill et al., 2018). In the context of retinitis pigmentosa, the oxidative DNA damage, mutations in antioxidant genes, and the upregulation of P2 × 7R are among the factors that regulate microglial activation and photoreceptor degeneration (Gallenga et al., 2021). Microglial cells migrate to phagocytize debris of dead cells, but the release of cytotoxic factors can kill adjacent cells (Gupta et al., 2003). In rd10 mice, there is an early presentation of “eat-me” signals on mutated rods, with an early infiltration of phagocytic microglia, pointing to microglial activation as an early contributor to cell death (Zhao et al., 2015). Yu et al. proposed that damaged photoreceptors attract microglia to the subretinal space to confer neuroprotection, whereas monocyte-derived cells accelerate the degeneration (Yu et al., 2020). Microglial activation has also been described in other IRDs, such as in Crumbs homolog 1 (CRB1)-associated retinopathies, which comprise a group of heterogeneous diseases including autosomal recessive RP type 12 (RP12), Leber congenital amaurosis type 8, cone-rod dystrophies isolated macular dystrophy, and X-linked foveal retinoschisis (Talib et al., 2017; Alves and Wijnholds, 2019). While the role of microglia cells in these diseases is mostly unknown, CRB1 models show an increased number of activated microglial cells in the outer nuclear layer and in the inner/outer segments layer (Alves and Wijnholds, 2019). Also, *Crb2* mutations result in a progressive retinal degeneration similar to RP, and *Crb2*-deficient mice show gliosis and microglial activation (Alves et al., 2013). IMPG2 (interphotoreceptor matrix proteoglycan) mutation, a mouse model of RP, also shows activated microglia invading photoreceptor layers and the subretinal lesions cause by IMPG1 proteoglycan accumulation (Salido and Ramamurthy, 2020). An increased number of microglial cells and signs of activation have also been detected in animal models of other IRDs: (i) choroideremia, a progressive X-linked degeneration of photoreceptors, RPE, and choroid (Tolmachova et al., 2010); (ii) a mouse model of congenital stationary night blindness (Leinonen et al., 2019); and (iii) a mouse model of Stargardt disease and macular degeneration (Kohno et al., 2013).

Due to this dual role of microglia in the degenerative process, the use of drugs to inhibit microglial activation in neurodegenerative diseases remains controversial. Fully blocking microglial activity is not a good therapeutic option, because they would lose their neuroprotective effects. Partial inhibition of their activation, influencing secreted pro-inflammatory mediators, could possibly limit the disease progression. This effect is expected to be greater than that obtained by the blocking of single cytokines (Karlstetter et al., 2015). The administration of several anti-inflammatory agents that prevent or reduce microglial activation has been proven to have neuroprotective effects in mouse models of IRDs. For example, the use of dexamethasone (a corticosteroid) in rd10 mice at the maximum peak of photoreceptors death partially preserved cone-mediated vision reducing retinal inflammation (Guadagni et al., 2019). Likewise, the progesterone analog norgestrel has shown anti-inflammatory and neuroprotective effects in rd10 mice by decreasing gliosis *via* its actions on microglia and Müller cells (Roche et al., 2018). The natural products such as curcumin (Wang et al., 2017) and apigenin (Chumsakul et al., 2020) have also been demonstrated to slow retinal degeneration in both rd10 and rd1 mice, at least in part by inhibiting microglial activation. Knowing the exact role and the multiple factors that affect microglial activation and its consequences in retinal neurodegenerative diseases will potentially provide new therapeutic targets.

#### Retinal macroglia: Astrocytes and Müller cells

In the healthy retina, astrocytes and Müller cells have specific roles to maintain the normal functioning of retinal neurons (Figure 2). Müller cells, the largest macroglial cells in retina, are distributed in tight contact with all retinal cells and perform an assortment of functions in healthy retina to maintain homeostasis. These functions include secreting a wide range of neurotrophic factors, aiding with the uptake and clearance of neurotransmitters as well as regulating the ion balance and pH in the tissue (Bringmann et al., 2006, 2009; Cuenca et al., 2014; Vecino et al., 2016; Devoldere et al., 2019; Reichenbach and Bringmann, 2020). Astrocytes are distributed in the most inner part of the retina, between the retinal nerve fiber layer and the ganglion cell layer, in close contact with blood vessels. These cells are star-shaped with long processes that interact with both neurons and blood vessels. Astrocytes are only present in vascularized retinas and are responsible not only for the development and preservation of retinal vessels but also for the inner blood-retinal barrier maintenance (Coorey et al., 2012; Cuenca et al., 2014). In a scenario of retinal injury or retinal alteration, as occurs in retinal dystrophies, glial cells undergo gliosis. This process seems to be different throughout the diseased retina. Human retinas show a varied gene expression profile of Müller and astrocyte cells depending on the localization of the retinal damage (Voigt et al., 2020). In a type of autoimmune retinopathy primarily affecting rod-photoreceptor cells, Müller and astrocytes cells from the periphery exhibit higher expression of gliosis-related genes than cells from central retina (Voigt et al., 2020).

The precise role of gliosis remains controversial. It is assumed that conservative or non-proliferative gliosis aims to protect neurons and to re-establish retinal homeostasis. Under persistent injury, however, the process can develop into a proliferative chronic gliosis that can be detrimental, exacerbating retinal inflammation, and neuronal death, ultimately forming a glial scar that diminishes the options for the tissue to recover. Increasing evidence indicates that the dual effects of gliosis can be related to the breakdown of the blood-retinal barrier or to an excessive pro-inflammatory response from microglial cells (Wang and Wong, 2014; Devoldere et al., 2019; Reichenbach and Bringmann, 2020). Thus, a better understanding of the interactions between these cells will be crucial for the development of future therapies.

##### Müller cells

Müller cells cover the entire retina, establishing tight contact with all other cells in the tissue, allowing them to react quickly to neuronal injury. Notably, Müller cells are highly resistant to pathogenic stimuli, owing to their high energy reserve and antioxidant capacity (Bringmann et al., 2009; Reichenbach and Bringmann, 2020). The normal function of Müller cells can be disrupted during retinal degeneration. For example, during photoreceptor cell loss in dystrophic RCS rats, Müller cells show loss of protein expression of aquaporin-4 and Kir4.1 channels, both involved in potassium and water homeostasis (Lassiale et al., 2016). Glutamate uptake can also be compromised (Charles-Messance et al., 2020).

A main feature of Müller cells in regard to gliosis is their high sensitivity to retinal damage. Indeed, Müller cell activation can be detected in retinal disease before neuronal cell death is evident. The first non-specific reaction to injury that has been described in these cells is the upregulation of intermediate filament proteins, such as nestin, vimentin, and glial fibrillary acidic protein (GFAP), which appears to be necessary for many of the responses of these cells to injury (Roesch et al., 2012; Valamanesh et al., 2013; Cuenca et al., 2014; Devoldere et al., 2019; Voigt et al., 2020). It seems that this reaction is not mediated by microglia, as the response is not triggered when microglia are selectively activated by lipopolysaccharide (LPS) injection in the P23H rat model of RP (Wang et al., 2011; Noailles et al., 2018).

Other non-specific responses of Müller cells to retinal injury include hypertrophy, migration, and proliferation. Müller cells of lower vertebrates have been proposed to have properties of stem/progenitor cells (Devoldere et al., 2019; Reichenbach and Bringmann, 2020). However, this has not been demonstrated in the mammalian retina, where the most common reaction of Müller cells to retinal damage is hypertrophy and formation of a glial scars (Cuenca et al., 2014; Devoldere et al., 2019; Reichenbach and Bringmann, 2020). Glial scar formation during photoreceptor cell loss has been associated with Müller cell reprogramming into an epithelial cell lineage mediated by transforming growth factor-β (TGFβ1/2) and Notch1/2 interactions *via* Smad3. In fact, blockage of Smad3 in a mouse model of light-induced retinal degeneration drives cell cycle re-entry in Müller cells to generate new neurons (Conedera et al., 2021).

Müller cells release a variety of growth factors, such as neurotrophins, b-FGF, pigment epithelium-derived factor (PEDF), GDNF, and leukemia inhibitory factor (LIF), among others, which can boost the survival of photoreceptor cells (Wang et al., 2011; Devoldere et al., 2019), but Müller cells also secrete pro-inflammatory molecules, such as IL-1β and IL-6, and induce the expression of an inducible nitric oxide synthase (iNOS), which together stimulate the inflammatory response (Wang et al., 2011). Hence, once again, the beneficial or harmful effects of gliosis are controversial.

There is strong evidence for coordinated Müller cell-microglia crosstalk during retinal degeneration in models of RP (Wang et al., 2011; Arroba et al., 2014; Roche et al., 2018). Müller cells act as antigen-presenting and immunocompetent cells in retinal disease (Bringmann et al., 2006). Indeed, it has been described that Müller cells are responsible for the majority of phagocytosis of dying rod photoreceptors, and they collaborate with microglia during the massive loss of these cells in the first stages of the disease (Sakami et al., 2019). It is also widely accepted that Müller cells coordinate the migration and activation of microglial cells during the course of degeneration through the expression of different biomolecules. Expression of the pro-inflammatory cytokine IL-1β from microglia promotes the expression of other chemokines by Müller cells, increasing the activation of the inflammasome during retinal degeneration (Natoli et al., 2017), and disrupting glutamate homeostasis by reducing the clearance of this neurotransmitter by Müller cells, thus enhancing photoreceptor cell death (Charles-Messance et al., 2020). Moreover, Müller cells modulate the expression of microglial adhesion molecules that mediate microglial recruitment to sites of damage (Bringmann et al., 2009; Wang et al., 2011; Reichenbach and Bringmann, 2020). Müller cells regulate the secretion of microglial chemoattractants such as CX3CL1, which induces an increase in the expression of CX3CR1 in microglia, enhancing microglia translocation to the inflammation area (Zhang et al., 2018). In addition, an analysis of the transcriptome from different cell types, including Müller cells, in mouse models of retinal diseases, showed that the latter upregulate the expression of different complement-activating components and downregulate the expression of complement inhibitors, which ultimately enhances microglial activation (Pauly et al., 2019; Jabri et al., 2020; Enzbrenner et al., 2021). Overall, these studies support the notion that Müller and microglial cells have closely coordinated functions and actively participate in retinal remodeling associated with the progression of the disease. In this context, rearrangement of cone photoreceptors, Müller cells, and microglia has been described in different models of RP when rod photoreceptors are almost completely lost (Lee et al., 2011; Fernández-Sánchez et al., 2015; Di Pierdomenico et al., 2020).

##### Astrocytes

Astrocytes are the main cell type responsible for the development and maintenance of the retinal vasculature, the blood-retinal barrier, and the blood flow. Astrocytes also collaborate with Müller cells and microglia in maintaining ionic homeostasis, neurotransmitter clearance, synaptic formation, and even in modulating synaptic transmission. In the context of retinal damage, astrocytes proliferate, migrate, and protect neuronal cells by releasing neurotrophic factors and restoring the blood-retinal barrier. Similar to Müller cell gliosis, astrogliosis has been associated with most of the retinal disorders, and different studies indicate that they can be both detrimental and neuroprotective for neuronal cells [reviewed in Cuenca et al., 2014; de Hoz et al., 2016; Vecino et al., 2016; Reichenbach and Bringmann, 2020] (Figure 2). In animal models of retinal dystrophies, such as RP, hypertrophy and hyperplasia of astrocytes have been associated with disease progression (Milam et al., 1998; Fernández-Sánchez et al., 2015). An increase in the expression of astrocyte GFAP in patients with rod photoreceptor degeneration caused by autoimmune retinopathy is a hallmark of astrogliosis, together with elevated expression of the astrocyte-specific inflammatory cytokine IFITM3, indicating intercellular communication between microglia and astrocytes (Voigt et al., 2020). The dual effects of astrogliosis have been attributed to the presence of two distinctive phenotypes of gliotic astrocytes in the brain (Escartin et al., 2019). However, further research is needed to clarify the role of astrogliosis in IRDs.

#### Changes in microglia, Müller cells, and astrocytes in inherited retinal dystrophies

**FIGURE 6:**
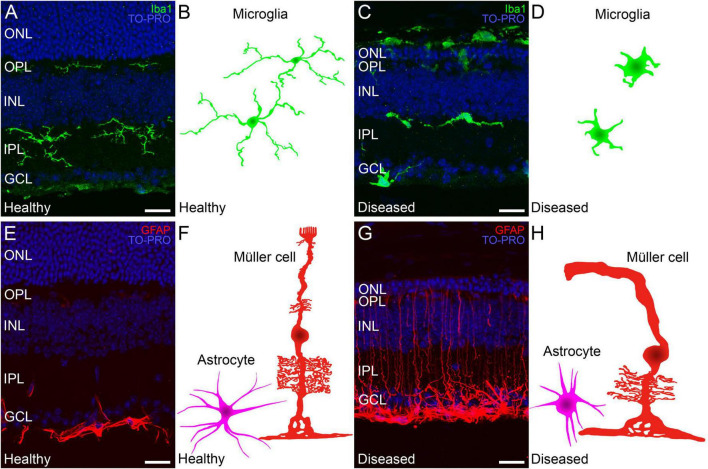
Changes in Müller cells and microglia during retinal degeneration. Retinal cross-sections immunostained with different retinal glial cell markers followed by a schematic drawing of each cell in a healthy mouse retina and in a retina from an IRD model. **(A,C)** Microglia immunostained against Iba1 (in green) and TO-PRO 3 iodide-stained nuclei (in blue). **(A)** In healthy retinas microglial morphology is ramified and cells are localized in the IPL, OPL and GCL. **(C)** Ameboid morphology from active microglia in a retina with an IRD. **(B,D)** Schematic depiction of the morphology of microglial cells in healthy **(B)** and diseased conditions **(D)**. **(E)** In healthy retinas GFAP (red) marker selectively immunostains astrocytes in the GCL. **(G)** In IRDs, astrocytes display a hypertrophied morphology and Müller cell activation causes increased GFAP immunoreactivity throughout the cell length. **(F,H)** Schematic depictions of representative Müller cells and astrocytes in healthy conditions **(F)** and affected by retinal degeneration **(H)**. ONL: outer nuclear layer; OPL: outer plexiform layer; INL: inner nuclear layer; IPL: inner plexiform layer; GCL: ganglion cell layer. Scale bars: 20 μm.

The primary death of rod photoreceptors in neurodegenerative diseases leads to subretinal accumulation and subsequent activation of microglia. Activated microglia transition from a ramified to an amoeboid morphology (Figures 1, 6A–D) (Noailles et al., 2014, 2016) and whereas they contribute to the phagocytotic clearance of rod cell debris, they also participate in the phagocytosis of viable photoreceptors and secretion of pro-inflammatory cytokines, potentiating photoreceptor apoptosis (Zabel et al., 2016; Rashid et al., 2019). In early retinal remodeling, Müller cells are among the first cell populations to display metabolic and morphological changes in response to stress. In addition to the thickening of the cell processes, they also show the signs of reactive gliosis such as increased expression of GFAP (Figures 1, 6E–H) (Cuenca et al., 2014). Further along the degenerative process, they undergo substantial hypertrophy and neighboring Müller cells merge into large radial glial columns (Pfeiffer et al., 2019). Astrocytes become reactive during this process and show typical hypertrophic morphology (Figures 1, 6E–H). In addition, the distal scaffolding of Müller cells collapses owing to the loss of photoreceptor cells, to form a distal seal that isolates the neural retina from the RPE and choroid (Jones et al., 2016).

Finally, changes in retinal vessel network have also been described (Cuenca et al., 2014). The gaps in the glial seal of the retina allow for the migration of choroidal vessels into the neural retina, often accompanied with RPE cells, displacing inner nuclear layer cells and forming migration columns (Cuenca et al., 2014). A disruption of the deep capillary plexus has also been observed in animal models of RP, with the loss of capillary density and capillary loops, hindering the normal supply of oxygen and nutrients to retinal cells (Fernández-Sánchez et al., 2018).

## Conclusion

Inherited retinal dystrophies have different origin and pathogenesis but share common mechanisms of cell death at molecular level. The oxidative stress, the high retinal sensitivity to oxidation, together with the increasing inflammation present in retinal tissue, leads to a progressive death of photoreceptors first, and progressively of the rest of the retinal cells (Cuenca et al., 2014, 2020). This cell death triggers tissue changes in an attempt to maintain functionality searching for new synaptic connections. Neurons, but also macro- and microglial cells and RPE, suffer a transformation. This transformation includes the changes in cell morphology, metabolism, protein expression, and network topologies. With the time, these changes lead to both, a loss of vision and to the progressive worsening of non-visual retinal functions, as the control of circadian rhythms and pupil contraction, caused by the degeneration of intrinsically photosensitive retinal ganglion cells (ipRGCs) (Esquiva et al., 2013; Lax et al., 2016). Non-visual retinal functions have also effects on memory and depression (Figueiro et al., 2018) and hence deeply influence the patient’s quality of life.

To date, there is no efficacious treatment against the etiologic cause of IRD. Hopes are high for gene- and cell-based therapies that could prevent, stop, or even revert the degenerative process in a more or less near future. Since then, a deep knowledge of the molecular mechanisms involved in retinal degeneration will hopefully show us suitable targets for the development of therapeutic molecules, mainly related to relevant players of the oxidative and inflammatory environment. It is likely that the use of antioxidant therapy and neurotrophic factors is to be necessary even when cell transplant and gene replacement therapies are available, as the success of these techniques will depend on the healthy environment of the retinal tissue, which would probably need the use of antioxidants or neuroprotectants. On the other hand, a deep knowledge of the changes associated with neurodegeneration in IRDs will help us to achieve success applying these therapies, as it will let us know the most suitable time frame to perform the transplant considering the phase of retinal degeneration, or which is the right place to place the cells, to get a proper migration and integration of the transplanted cells inside the retinal tissue.

## Author contributions

IP, VM, NM-G, and NC: conceptualization. IP, VM, LC, and NM-G: methodology. OK, XS-S, CS-C, and LF-S: investigation. LF-S and PL: resources and writing—original draft preparation. IP, VM, NM-G, LC, NC, and PL: writing—review and editing. IP and NC: funding acquisition. All authors have read and agreed to the published version of the manuscript.
